# Spatiotemporal evolution of innovation collaboration networks in China’s AI medical device industry: implications for public health governance

**DOI:** 10.3389/fpubh.2026.1899325

**Published:** 2026-08-19

**Authors:** Feng Hu, Huijie Yang, Junyu Cheng, Zhijie Huang, Shaobo Yang, Xiaoping Wang, Haiyan Zhou, Jiahan Hu, Hao Hu, Shuang Zhao

**Affiliations:** 1 Institute of International Business & Economics Innovation and Governance, Shanghai University of International Business and Economics, Shanghai, China; 2 Industrial Technology Research Center, Yice Research Institute, Shanghai, China; 3 International Business School, Shanghai University of International Business and Economics, Shanghai, China; 4 Elliott School of International Affairs, The George Washington University, Washington, DC, United States; 5 College of Management, Ningbo University of Finance & Economics, Ningbo, China; 6 Graduate School, Nueva Ecija University of Science and Technology, Cabanatuan, Philippines; 7 College of Engineering, University of Perpetual Help System Laguna, Biñan, Laguna, Philippines; 8 School of Economics, Shanghai University, Shanghai, China

**Keywords:** AI medical devices, innovation network, public health, regional health inequality, Yangtze River Delta

## Abstract

Artificial intelligence (AI) medical devices have become an important technological foundation for enhancing healthcare system resilience and promoting the equitable allocation of medical innovation resources. However, limited attention has been paid to the distribution of upstream innovation resources and the spatial organization of this industry in the wake of the pandemic. Using AI medical device patent collaboration data from the Yangtze River Delta during 2018–2025, this study constructs local and external collaborative innovation networks and applies social network analysis and the Geodetector to examine their spatial evolution and associated factors across different phases of the COVID-19 pandemic. The results show that all innovation networks expanded continuously, particularly the external collaborative network. Nevertheless, cross-regional collaboration remained concentrated in a limited number of core cities, resulting in a persistent core–periphery structure. Regional GDP, retail market size, higher education resources, science expenditure, and financial support consistently exhibited strong explanatory power for collaboration intensity, while GDP growth and openness became increasingly associated with network differentiation following the COVID-19 shock. Moreover, the explanatory power of most variables was stronger for external collaboration than for local collaboration, suggesting that cross-regional knowledge exchange relies more heavily on comprehensive innovation capacity. This study advances the understanding of the spatial organization of AI medical innovation and provides empirical evidence for promoting a more balanced allocation of cross-regional medical R&D resources.

## 1 Introduction

Artificial intelligence (AI) medical devices have become an important component of digital health systems and an emerging driver of public health innovation (1). Given the dual backdrop of the in-depth advancement of the “Healthy China 2030” strategy and accelerating population aging, the application value of AI medical devices in public health scenarios such as disease screening, assisted diagnosis, and remote health data monitoring is becoming increasingly prominent. In recent years, the National Medical Products Administration (NMPA) of China has continuously expanded its review and approval procedures, with the number of approvals for AI medical devices and advanced medical imaging equipment in key fields continuously increasing. For example, in March 2020, the NMPA of China issued the *Guidelines for the Review of CT Image-assisted Triage and Evaluation Software for Pneumonia (Trial)*, which accelerated the regulatory review and approval of AI-powered pneumonia diagnostic products. In April 2021, the pneumonia CT image-assisted triage and evaluation software developed by Deepwise Medical and Infervision became the first AI-based pneumonia products to receive NMPA approval in China, marking a significant milestone in the clinical translation of AI medical technologies.

By the end of 2025, the five provinces and municipalities of Beijing, Shanghai, Jiangsu, Guangdong, and Zhejiang collectively accounted for more than 70% of the national approved innovative medical device products. Furthermore, thanks to its strong industrial foundation, high level of innovation, and concentrated scientific and educational resources, the Yangtze River Delta (YRD) is rapidly emerging as a core agglomeration area for China’s advanced medical device innovation,^1^ and its applications in public health settings are also at the forefront nationwide. In early 2020, Shanghai Public Health Clinical Center guided Yitu Medical in developing an “Intelligent Assessment System for Novel Coronavirus Pneumonia,” which can rapidly complete quantitative analysis and efficacy evaluation of lung lesions in CT images, with the assessment results showing high consistency with expert evaluations.^2^ In 2018, IDX-DR, the first AI medical device for diabetic retinopathy screening, received FDA approval in the United States, marking the industry’s entry into commercial application. That same year, the China Medical Equipment AI Alliance, aimed at promoting deep integration of AI and clinical medicine, was established in Suzhou, signaling the industry’s entry into a professionalized trajectory. Consequently, while the industry has now established a mature industrial system and a market-oriented development framework, there remains a lack of systematic research on the patterns of technology diffusion and the mechanisms governing the cross-regional transfer of R&D resources within this industry.

The outbreak of COVID-19 has prompted scholars around the world to become more actively engaged in research on how to build more resilient and responsive public health systems. Lee et al. (2) reviewed the literature about public health systems and summarized that topics such as collaborative networks, system resilience, health equity, prevention, and decision-making capacity have garnered the most attention from scholars over the past decade. Meanwhile, both Haldane et al. (3) and Rawat et al. (4) proposed that system preparedness, response speed, cross-sectoral engagement, and care capacity are key indicators for evaluating healthcare systems. Olawade et al. (5) and Panteli et al. (6) further explored the practical applications and potential issues of AI in public health within the context of emerging technologies. The inherent characteristics of AI-enabled industries, such as resource integration capabilities, efficiency, and accuracy, have made the role of AI medical devices increasingly prominent in responding to public health emergencies and improving the quality of primary public health services (7–10). Existing studies can be broadly classified into three categories that regulatory and approval circumstances (11, 12), the interrelationship between AI and medical devices, such as AI applications in medical devices (1, 13, 14), and how AI improves medical device utilization efficiency (15) without fully addressing the evolutionary trends of the AI medical device industry itself or the influencing forces of innovation in its upstream sectors.

A deeper division of labor within the market drives innovation activities among different actors and across regions (16). Innovation networks, as important carriers of knowledge flow and technology diffusion, have become a core subject in innovation geography and regional economics (17–20). Research on innovation networks has demonstrated a clear trajectory from microspatial subjects to macrospatial scales. Early studies predominantly focused on micro-organizational levels such as enterprises, universities, and research institutions, exploring the conceptual connotations and collaborative innovation models of innovation networks (21, 22). With the deepening of globalization and the development of the knowledge economy, research scales have progressively expanded to cities, urban agglomerations, national systems, and even global levels, with cities’ roles as nodes in innovation networks and the functional division of labor gradually becoming research hotspots (23–34).

Social network analysis methods are commonly used to analyze network structural characteristics. Scholars conduct systematic analyses at three levels: overall network attributes, node location characteristics, and types of node relationships. They typically use objective and easily collectible data, such as patent collaborations and patent transfers, to construct innovation networks (35–39). Existed findings reveal that innovation networks typically exhibit small-world and scale-free properties, with core nodes playing important roles as “gatekeepers” and “bridges” (40–50). Early research by Lissoni, Llerena & Sanditov (42) suggested that the more universities and research institutes participate in technology cooperation networks, the more pronounced the small-world characteristics become. Losacker (44) pointed out that the diffusion of green technologies in China is largely dominated by a few regions and constrained by regional reciprocal relationships, whereas R&D in the lead mining industry can overcome geographical constraints and is driven by demand from distant regions. Furthermore, both Genin et al. (51) and Cai et al. (52), who focused on China’s high-end capital-intensive manufacturing industries, revealed that structures dominated by a few leading hubs may be more efficient in terms of R&D than dispersed, dense networks. With respect to influencing factors of networks, scholars have primarily employed multidimensional proximity theory and have discovered that geographical proximity, cognitive proximity, social proximity, and institutional proximity significantly influence the formation of innovation linkages. Macro factors such as regional economic development levels, government scientific and technological investment, and industrial policy orientation also demonstrate significant explanatory power (53–63). As a capital- and knowledge-intensive strategic industry, AI medical device innovation relies on intensive collaboration among hospitals, universities, research institutions, and enterprises. Unlike conventional manufacturing industries, its innovation activities are simultaneously embedded in industrial innovation systems and public health systems, making the spatial organization of innovation resources crucial for both technological competitiveness and the accessibility of AI-enabled healthcare services. Given the substantial disparities among Chinese cities in economic development, innovation capacity, healthcare infrastructure, and higher education resources, the formation and evolution of innovation networks are inherently shaped by multidimensional spatial factors. These characteristics suggest that integrating network analysis with spatial explanatory approaches provides an appropriate analytical framework for investigating the formation and evolution of AI medical device innovation networks.

Overall, existing studies have generated substantial insights into the AI medical device industry. However, important gaps remain in understanding its development from a macro-level industrial perspective. First, regarding the research focus, previous studies have predominantly examined issues such as the effectiveness of AI applications, supporting ecosystems, and policy frameworks. However, the AI medical device industry cannot be fully understood by focusing solely on localized collaboration or specific application scenarios. Such perspectives fail to capture how upstream innovation resources circulate and interact across the entire industrial system. Second, with respect to the scale of innovation network analysis, existing studies have largely concentrated on either national-level or regional-level networks in isolation. Few studies have incorporated intra-regional collaboration and interregional spatial spillovers into a unified analytical framework. Third, most existing research has focused on the routine spatial evolution of innovation networks under normal development conditions. Comparatively little attention has been paid to how innovation networks respond to major external shocks, such as the COVID-19 pandemic and other large-scale public health emergencies. Finally, as the heterogeneous core of innovation activities, the micro-level behavior of innovation actors directly determines the evolution of macro-level networks. Existing research has failed to effectively elucidate the intrinsic coupling mechanisms between the two, and thus has not been able to uncover richer structural information. Given this context, this study analyzes the spatial evolution of the technological innovation network in the AI medical device industry in the YRD from 2018 to 2025, revealing its inherent evolutionary laws and influencing mechanisms. This finding holds important theoretical value and practical significance for optimizing the allocation of regional innovation resources, enhancing the capacity to respond to public health emergencies, and reducing regional disparities in public health innovation capacity.

## 2 Research methodology and data sources

### 2.1 Research data

First, data were retrieved from the Incopat global patent database using the following search query: MKCLAS2 = (502) AND INDUSTRY2 = (1.5). This targets the medical device manufacturing industry (502) with an emerging industry classification of AI (1.5). By filtering for Chinese patents with more than one applicant obtained between 2018 and 2025, a total of 14,820 relevant patents were identified. Second, the data were cleaned and organized. Patent applicants were extracted, and their registered provinces and cities were queried in batches via the Aiqicha website. After entries with unclear addresses and patents involving collaboration within the same city were removed, a final set of 1,637 relevant patents was obtained. Excluding intra-city collaborative patents allows the research to focus on the spatial linkages between inter-city flows of innovation resources and knowledge diffusion. Compared to inter-city collaboration, intra-city collaboration exhibits greater consistency in institutional environments, public health governance capabilities, and conditions for innovation, and the organizational characteristics of collaborative relationships are more pronounced. Including such collaborations in the analysis could easily weaken or even obscure the spatial differences in inter-regional innovation collaboration, thereby hindering the identification of patterns in the cross-regional allocation of innovation resources. Third, a city collaboration innovation matrix was established. If a single patent involves three cities (A, B, and C), it is recorded as having three distinct collaborative innovation links: A-B, A-C, and B-C. These were aggregated to form the final matrix. Fourth, in this paper, Local Network refers to the collaborative innovation network among cities within the YRD. The External Collaborative Network refers to the collaborative innovation network between YRD cities and cities outside the region. The Two-Layer Network is the summary of both the local and external collaborative networks. The YRD was selected as the analytical unit for the local network for the following reasons. Firstly, as a nationally designated integrated development region, the YRD has witnessed increasing industrial collaboration, innovation resource flows, and scientific cooperation that transcend provincial administrative boundaries.^3^ Intercity innovation linkages are increasingly characterized by functional specialization and collaborative complementarities, providing a representative case for the development of an integrated regional innovation system. Accordingly, defining collaborations between YRD cities and cities outside the region as the external collaborative network facilitates the examination of how administrative boundaries and geographical distance constrain innovation collaboration, while revealing knowledge diffusion dynamics between innovation core areas and peripheral regions. Secondly, the YRD is one of China’s most concentrated regions for healthcare resources and the biopharmaceutical industry, and it also serves as an important pilot region for coordinated public health governance. Examining knowledge exchanges in the external collaborative network helps improve our understanding of how innovation resources diffuse across regions under conditions of uneven healthcare resource distribution. Finally, to avoid anomalies caused by specific years, the research period was divided into four phases: 2018–2019, 2020–2021, 2022–2023, and 2024–2025.

### 2.2 Research methods

#### 2.2.1 Social network analysis

Using Gephi software, an analysis of network density, average degree, average path length, and weighted degree centrality was conducted for the local, external collaborative, and two-layer networks of the AI medical device industry in the YRD (64–66). In this paper, Internal Collaboration Innovation Intensity refers to the weighted degree centrality of YRD cities within the Local Network, whereas External Collaborative Innovation Intensity refers to their weighted degree centrality within the external collaborative network.

#### 2.2.2 Geographical detector (Geodetector)

Compared with conventional regression-based approaches, the Geodetector can directly measure spatial stratified heterogeneity without requiring assumptions of linearity or normality, and it is capable of identifying nonlinear interaction effects among explanatory variables. To reduce short-term fluctuations and construct more stable innovation networks, patent collaboration data were aggregated into three developmental stages: 2018–2019, 2020–2021, and 2022–2023, representing the pre-pandemic, pandemic, and period after pandemic, respectively. This stage-based design facilitates comparisons of the evolution of innovation networks and their explanatory mechanisms under different public health contexts while ensuring sufficient network density for reliable Geodetector analysis. Before conducting the Geodetector analysis, both the dependent and independent variables were discretized using the Natural Breaks (Jenks) classification method in ArcGIS and categorized into five classes to construct the spatial stratification of intercity innovation collaboration intensity. Specifically, the external and internal collaborative innovation intensities of YRD cities from Phase I to Phase III were used as the dependent variables. Four dimensions, economic development, innovation factor support, fiscal and financial support, and opening-up levels, were analyzed. Factors such as the proportion of secondary and tertiary industry value in GDP, per capita GDP, and regional GDP were selected as influencing factors to examine the explanatory mechanisms of the innovation network (67–69). It should be noted that Geodetector identifies the explanatory power of spatial stratified heterogeneity rather than causal relationships. Therefore, the results should be interpreted as revealing the spatial explanatory mechanisms associated with the evolution of innovation networks rather than establishing strict causal effects.

## 3 Evolutionary patterns of innovation networks from a National-Local perspective

### 3.1 Basic attributes of the innovation network

From an overall network-scale perspective, the technological innovation network for the AI medical device industry in the YRD has shown a continuous expansion trend across the four time periods (Table 1). For the external collaborative network, local network, and two-layer network, both the number of nodes and the number of edges steadily increase. This indicates that a growing number of urban entities are engaging in AI medical device innovation and that collaborative innovation between cities is steadily increasing. Specifically, the two-layer network showed the most significant growth trend, with nodes increasing from 40 in Phase I to 123 in Phase IV, and the number of edges increasing from 68 to 360. This indicates that as more cities outside the region are incorporated into the cooperation framework, innovation resources in the YRD are gradually transcending regional boundaries, evolving from internal regional concentration to cross-regional diffusion. Notably, the growth rate of nodes in the external collaborative network has consistently outpaced that of the local network. By Phase IV, the number of nodes in the external collaborative network reached 115, far exceeding the 36 nodes in the local network. This implies that innovation activities in the YRD’s AI medical device field are now deeply embedded within the national-scale technological innovation collaboration system, which means the advanced medical R&D resources in the YRD can radiate outward, greatly contributing to balancing the health governance capacities across different regions. With respect to the evolution of topological features in the network structure, the average degree of the local network increased from 2.778 in Phase I to 6.111 in Phase IV, with the average weighted degree increasing to 42.5 in Phase IV. Network density also fluctuated from 0.163 to 0.175. These figures suggest that collaborative innovation among internal cities in the YRD is becoming increasingly tight, resulting in a clear improvement in regional innovation integration, and the medical R&D capacities across regions have become more synchronized. In contrast, while the average degree of the external collaborative network also tended to increase, the network density decreased from 0.081 to 0.038. This indicates that network expansion has outpaced the growth of collaborative ties. Innovation linkages remain concentrated around a limited number of core cities, with peripheral cities yet to establish stable collaborative relationships, resulting in a persistent core–periphery structure. Consequently, while the YRD has strengthened its role as a hub for cross-regional knowledge diffusion, innovation collaboration remains highly centralized. The clustering coefficient of the two-layer network increased from 0.443 to 0.684, whereas the average path length decreased from 2.615 to 2.389. This demonstrates that the “small-world” nature of the overall innovation network became more pronounced, leading to a continuous improvement in the efficiency of collaborative innovation for AI medical devices. This phenomenon holds important implications for healthcare governance. First, the improved efficiency of knowledge diffusion enables the accelerated deployment of digital medical technologies during public health crises. Second, the enhanced network connectivity has reduced structural barriers between core and peripheral regions, helping to mitigate regional disparities in healthcare innovation capacity. Third, the existence of key hub nodes underscores the importance of strategically supporting leading enterprises and hub cities, which is crucial for driving cross-regional collaboration.

**Table 1:** Network characteristics of urban innovation networks from a national-to-local perspective.

Network attributes	External collaborative network				Local network				Two-Layer network			
	P1	P2	P3	P4	P1	P2	P3	P4	P1	P2	P3	P4
Node	33	58	81	115	18	27	35	36	40	68	91	123
Edge	43	88	144	250	25	49	69	110	68	137	213	360
Average degree	2.606	3.034	3.556	4.348	2.778	3.63	3.943	6.111	3.4	4.029	4.681	5.854
Average weighted degree	8.909	12.379	15.506	22.052	15.222	13.778	13.657	42.5	14.2	16.029	19.055	33.057
Network diameter	6	6	7	5	5	5	4	4	5	5	5	4
Network density	0.081	0.053	0.044	0.038	0.163	0.14	0.116	0.175	0.087	0.06	0.052	0.048
Average clustering coefficient	0	0	0	0	0.5	0.633	0.635	0.697	0.443	0.567	0.601	0.684
Average path length	2.887	2.892	2.959	2.856	2.529	2.436	2.277	2.149	2.615	2.524	2.491	2.389

Overall, the three innovation networks exhibit a typical core–periphery structure. As the networks expanded, peripheral cities were gradually integrated into the regional innovation system, and the overall network evolved from a core city-dominated pattern toward a more open and multi-level collaborative structure. Nevertheless, the continuous decline in the density of the external collaborative network indicates that innovation resources remain concentrated in a limited number of core cities, suggesting that interregional knowledge diffusion still follows a pronounced hierarchical pattern. From a temporal perspective, the COVID-19 pandemic caused a temporary disruption to innovation collaboration within the YRD. However, as pandemic control entered the normalization stage and the post-pandemic period, nationwide innovation collaboration gradually recovered, further strengthening the YRD’s role as a regional innovation hub and enhancing its capacity for cross-regional knowledge diffusion.

### 3.2 Collaborative innovation intensity in cities outside the YRD

With respect to the innovation intensity of collaboration with cities outside the YRD, Beijing, Guangzhou, and Shenzhen have consistently remained in the first tier (Table 2). Beijing’s collaborative innovation intensity surged from 67 in Phase I to 495 in Phase IV, maintaining the top position with an absolute advantage. These findings indicate that innovation collaboration between the YRD and core cities in the Beijing-Tianjin-Hebei (BTH) in the field of AI medical devices is continuously deepening. This exceptionally high intensity did not significantly change across the various phases of the pandemic, demonstrating the stable radiation effect of Beijing as the national source of technological innovation. Guangzhou and Shenzhen alternately occupied the second and third positions. Their collaborative innovation intensity has also steadily increased, reaching 99 and 96, respectively, in Phase IV. This signifies that the technological collaboration and innovation links between the YRD and the Pearl River Delta (PRD) city cluster are becoming increasingly close. In addition, beyond first-tier cities, regional central cities such as Shenyang, Chengdu, Xi’an, and Tianjin have consistently maintained high rankings. Nonprovincial capital cities such as Wenzhou, Yantai, Zhuhai, and Foshan successively entered the top 20 in the later phases. This suggests that external innovation collaboration from the YRD is diffusing toward nonprovincial capital cities. This expanding pattern of cross-regional collaboration suggests that knowledge and R&D resources in the AI medical device industry are gradually diffusing from national innovation hubs to regional central cities and, increasingly, to non-capital cities. Although innovation collaboration remains concentrated in a limited number of core nodes, strengthening innovation linkages in peripheral cities lays the foundation for the wider dissemination of advanced medical technologies. It creates new opportunities to reduce regional disparities in public health technology capacity. The expansion model, from point to area, further extends the findings of Genin et al. (51) and Cai et al. (52), suggesting that innovation networks centered around a small number of hub nodes may outperform densely connected networks in terms of R&D efficiency.

**Table 2:** Collaborative innovation intensity in cities outside the YRD (top 20).

Rank	City	P1	City	P2	City	P3	City	P4
1	Beijing	67	Beijing	179	Beijing	260	Beijing	495
2	Guangzhou	25	Guangzhou	31	Shenzhen	36	Guangzhou	99
3	Shenzhen	14	Shenzhen	24	Shenyang	34	Shenzhen	96
4	Shenyang	10	Chengdu	19	Tianjin	27	Foshan	43
5	Zhengzhou	8	Wenzhou	13	Guangzhou	26	Chengdu	38
6	Xi’an	6	Yantai	13	Jinan	23	Xi’an	36
7	Changsha	3	Xi’an	10	Chengdu	22	Tianjin	32
8	Chengdu	2	Zhengzhou	10	Xi’an	22	Wuhan	31
9	Tianjin	2	Wuhan	8	Zhuhai	21	Chongqing	30
10	Chongqing	2	Tianjin	7	Zhengzhou	16	Wenzhou	28
11	Yinchuan	1	Zhuhai	5	Xiamen	11	Haikou	27
12	Harbin	1	Changsha	5	Qingdao	11	Xiamen	22
13	Jinan	1	Yinchuan	5	Nanchang	11	Jinan	21
14	Ji’an	1	Qingdao	4	Chongqing	10	Qingdao	21
15	Dongguan	1	Foshan	4	Changsha	10	Nanchang	18
16	Zhongshan	1	Harbin	4	Wuhan	8	Shenyang	17
17	Putian	1	Kunming	3	Foshan	6	Changsha	17
18	Datong	1	Fushun	3	Wenzhou	6	Zhengzhou	16
19			Jinan	2	Sanya	6	Linyi	13
20			Nanchang	2	Yantai	5	Fuzhou	12

### 3.3 Intensity of external collaborative innovation among YRD cities

**Figure 1:**
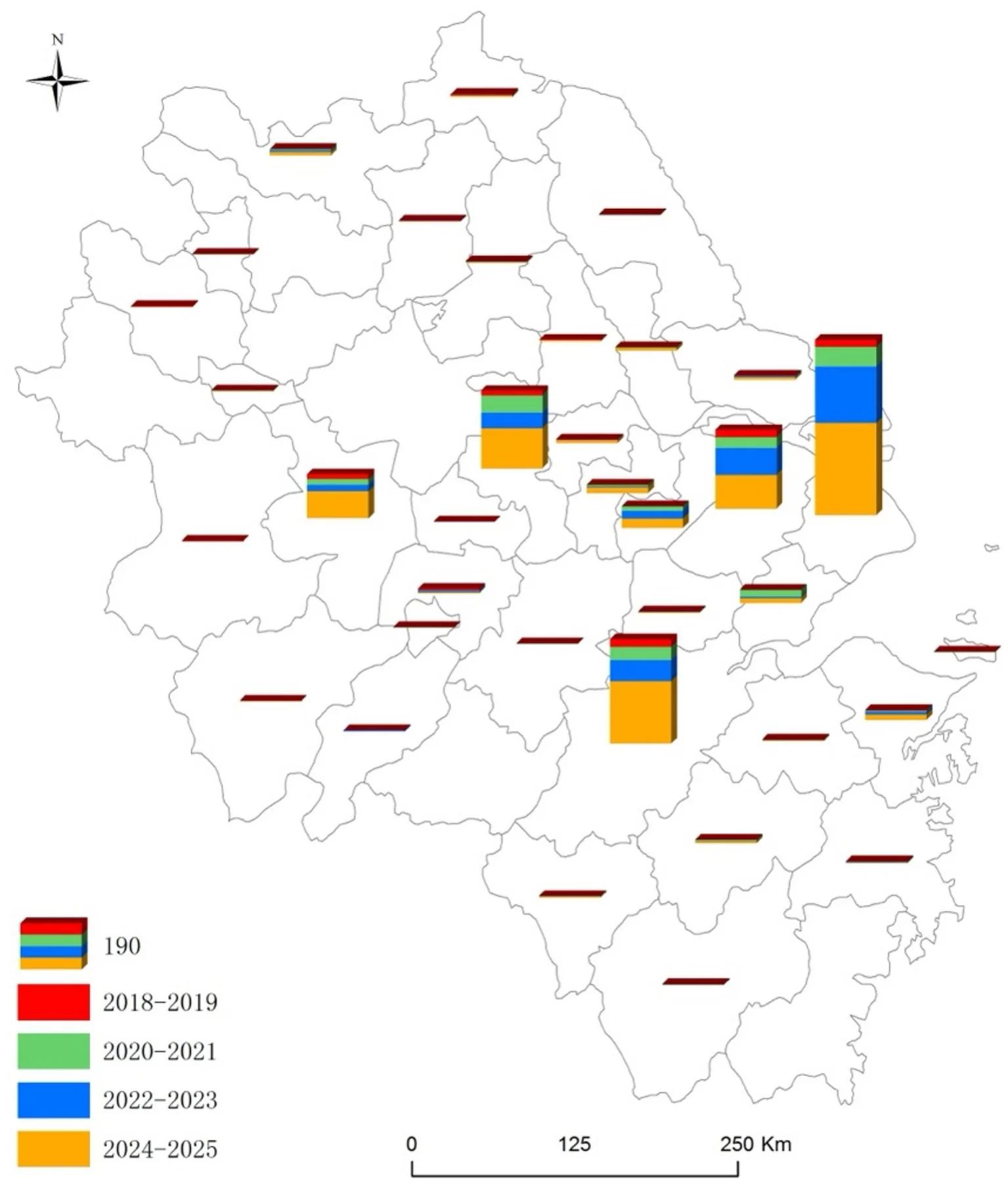
External collaborative innovation intensity of YRD cities.

From the perspective of the dynamic evolution of external collaborative innovation intensity for various cities of the YRD, as the core hub, Shanghai’s external collaborative innovation intensity rapidly increased from 29 in Phase I to 378 in Phase IV (Figure 1). Notably, it jumped to 233 in Phase III, far exceeding that of the other cities during that period. This finding indicates that Shanghai assumed the role of a core gateway for innovation collaboration between the YRD and external cities as early as the mid-pandemic period, a function that further expanded in the post-pandemic era. Hangzhou and Suzhou form the second tier. Hangzhou increased from 30 in Phase I to 257 in Phase IV, while Suzhou increased from 30 to 139. Both cities experienced significant increases during Phase III. Nanjing and Hefei are provincial capitals whose performance is eye-catching. Nanjing grew from 19 to 165, and Hefei grew from 20 to 111. Both cities exhibited growth characteristics that doubled by Phase IV.

The evolution of spatial distribution reveals that the intensity of external collaborative innovation is shifting from a concentration on a few early core cities to a diffusion trend among more small and medium-sized cities. In Phase I, only a few cities, such as Hangzhou, Shanghai, Suzhou, Nanjing, and Hefei, showed significant external collaborative innovation, and for most other cities, the intensity was zero or near zero. In Phase IV, cities such as Changzhou, Nantong, Ningbo, Xuzhou, and Zhenjiang experienced breakthrough growth. Specifically, Changzhou jumped from 0 to 25, Nantong increased from 1 to 11, and Xuzhou increased from 0 to 14. These findings indicate that cities in the northern YRD and central Jiangsu Province are gradually becoming embedded in the external collaborative innovation network. Furthermore, while cities within Anhui Province, such as Wuhu, Anqing, and Bozhou, have shown sporadic growth, their overall collaborative innovation intensity remains relatively weak. This suggests that the YRD integration strategy still faces certain administrative barriers regarding outward-looking innovation collaboration in the field of AI medical devices. This also indicates that formal policy frameworks alone are insufficient to ensure the effective conduct of collaboration. From a governance perspective, breaking down institutional barriers and reducing coordination costs are pivotal to driving the cross-regional diffusion of technologies. Furthermore, the limited participation of peripheral cities reflects the characteristic of their shallow network embedding, which also restricts the cultivation of local endogenous innovation capabilities. Therefore, deepening the depth of collaboration requires both expanding the scope of participation and enhancing the depth of collaborative engagement.

### 3.4 Internal collaborative innovation intensity of cities in the YRD

**Figure 2:**
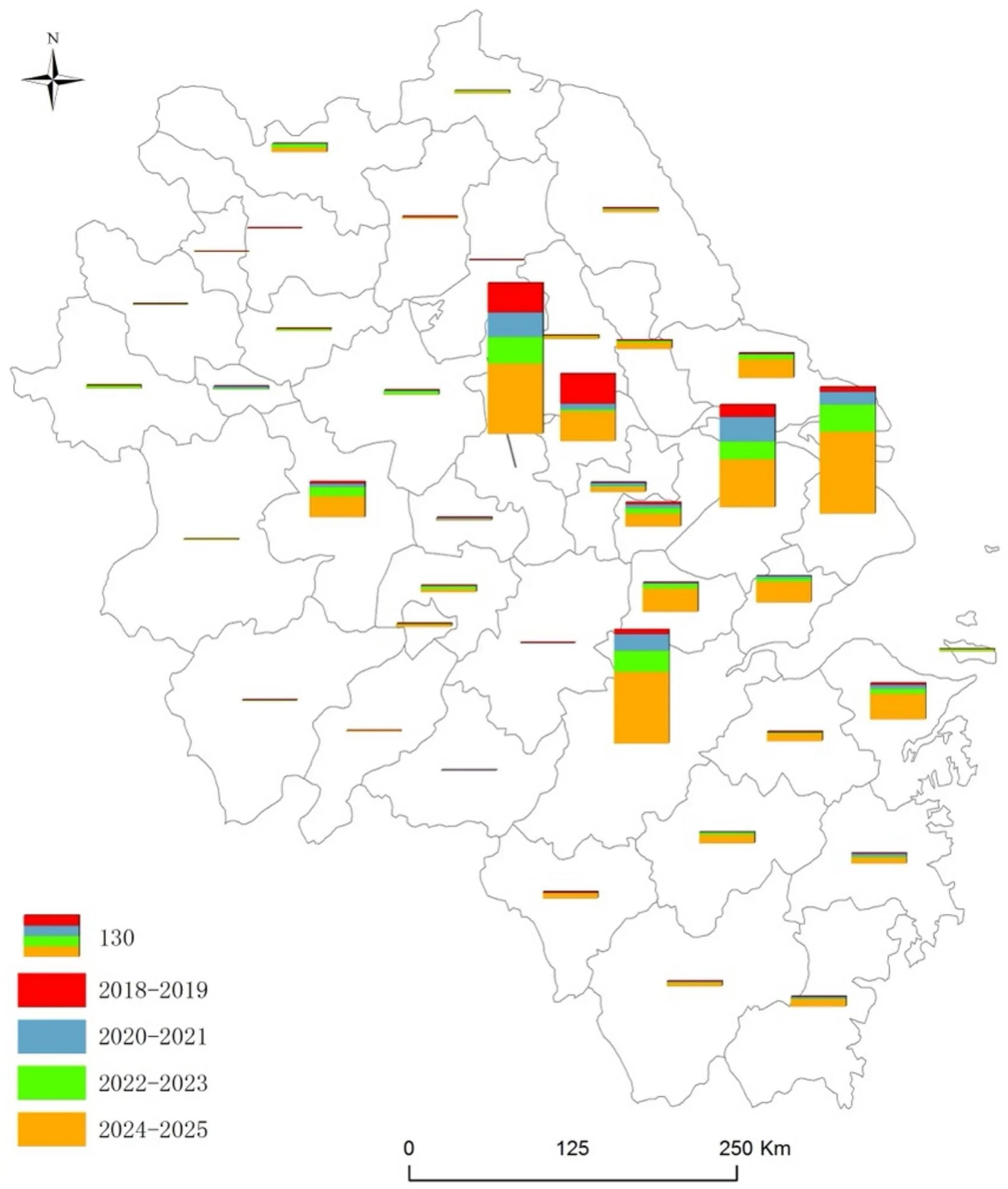
Internal collaborative innovation intensity of cities in the YRD.

In terms of the evolution of internal collaborative innovation intensity among cities in the YRD, four cities, Shanghai, Hangzhou, Nanjing, and Suzhou, have consistently maintained an absolute dominant position (Figure 2). In Phase IV, the internal collaborative innovation intensity of these cities skyrocketed: Shanghai 253, Hangzhou 219, Nanjing 216, and Suzhou 148. Together, these four cities account for more than 60% of the total internal collaborative innovation volume of all the cities, forming the core phalanx of the YRD’s AI medical device collaborative innovation network. In addition, Nanjing led with an intensity of 90 in Phase I. Although it was overtaken by Shanghai and Hangzhou during Phases II and III, it rebounded to 216 by Phase IV. This finding indicates that after a periodic adjustment during the early phases of the pandemic, Nanjing re-established its status as a hub for internal collaborative innovation in the YRD. Zhenjiang’s performance here was unique. In Phase I, its innovation intensity was high at 91, second only to Nanjing, but it plummeted to 19 in Phase II and 5 in Phase III before rebounding to 92 in Phase IV. This shows significant volatility.

With respect to the evolution of the spatial pattern, in Phase I, the internal collaborative innovation intensity of most cities, excluding Shanghai, Hangzhou, Nanjing, Suzhou, and Zhenjiang, was zero or near zero. However, by Phase IV, cities such as Ningbo, Huzhou, Hefei, Jiaxing, and Nantong all exceeded 50, with Huzhou rising from 0 to 71, Jiaxing from 0 to 63, and Nantong from 2 to 57. While the overall collaborative innovation intensity of cities in Anhui Province remains relatively low, that of Hefei improved from 5 to 63, and cities such as Wuhu, Chuzhou, and Ma’anshan performed in specific phases. Cities in northern Anhui and northern Jiangsu, such as Suzhou, Huaibei, and Huai’an, remained at extremely low levels even during Phase IV. The status of these peripheral cities in the northern YRD within the network urgently needs to be improved. Combined with the external collaboration intensity of YRD cities, the results indicate that both regional R&D activities and cross-regional knowledge diffusion remain highly concentrated in a few core cities. This suggests that the policy and locational advantages of the YRD integration strategy have not yet been fully transformed into a broad-based innovation collaboration network. According to the findings of Haldane et al. (3) and Rawat et al. (4), an effective public health emergency response system requires rapid response times and interdepartmental coordination; however, overreliance on a limited number of hubs for knowledge production and dissemination may hinder the nationwide diffusion of AI medical technologies, thereby limiting the technological support available for building a more balanced and resilient public health governance system.

### 3.5 Spatial pattern of external collaborative innovation networks for cities in the YRD

**Figure 3:**
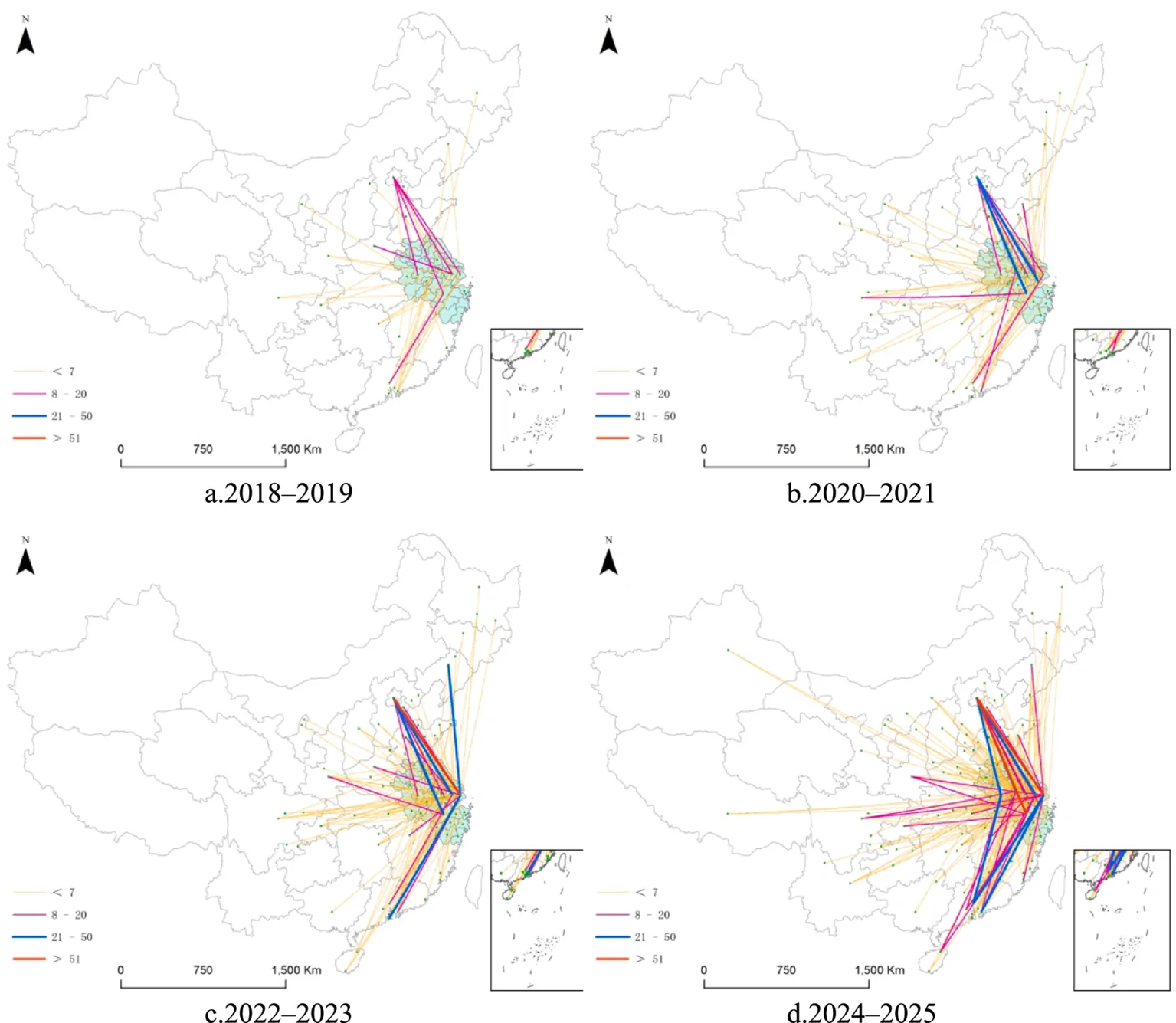
Spatial patterns of external collaborative innovation networks of the YRD.

From the perspective of the evolution of external collaborative innovation for cities in the YRD, Beijing, as the core of the national scientific and technological innovation center, has consistently maintained a stable position (Figure 3). In all four phases, it has been the closest external collaborative innovation partner for YRD cities, with a significant increase in innovation intensity. In Phase I, the collaborative innovation intensity with Hangzhou, Hefei, and Shanghai was only 14. By Phase IV, the Hangzhou–Beijing intensity reached 163, while that of Shanghai–Beijing reached 115. The collaborative intensity of Suzhou, Nanjing, and Hefei also exceeded 40, forming the main axis of the YRD external collaborative innovation network.

On the basis of the four-tier classification of collaborative innovation intensity in Phase IV, all collaborative innovation in the first tier is concentrated in Beijing and stems from collaboration among Hangzhou, Shanghai, and Suzhou. The spatial direction of collaboration in the second tier began to diversify. In addition to Beijing, cities in the PRD, such as Guangzhou, Shenzhen, and Foshan, appeared frequently. The formation of paths such as those in Hefei–Guangzhou (28), Hefei–Foshan (20), Shanghai–Shenzhen (40), and Shanghai–Guangzhou (33) signifies deepening collaboration between the YRD and PRD urban clusters in AI medical device technical innovation. Central cities in the central and western regions, such as Chengdu, Xi’an, and Haikou, also entered this tier. Collaborative innovation in the third tier further diffused to include cities such as Qingdao, Xiamen, Tianjin, Shenyang, Wuhan, and Chongqing. These cities cover urban clusters in the Shandong Peninsula, the west coast of the Taiwan Strait, BTH, the middle reaches of the Yangtze River, and the Chengdu–Chongqing region. Furthermore, the participating YRD cities are no longer limited to Shanghai and Hangzhou; Nanjing, Suzhou, Wuxi, and Jiaxing have also joined. Collaborative innovation in the fourth tier has spread across numerous cities throughout the eastern, central, and western parts of the country.

In summary, the four-tiered structure not only reflects the completeness of the network’s nationwide spatial coverage but also addresses diverse public health needs through differentiated approaches. In particular, the relatively low level of collaboration at the fourth tier reflects clear characteristics of spatial marginalization. In the future, efforts should be made to strengthen guidance on innovative collaboration targeting underdeveloped regions, bridge gaps in R&D capabilities through cross-regional technology spillovers, and ultimately achieve an equitable distribution of national public health emergency technology reserves.

Regarding the external collaboration network at the patent applicant level (Table 3), interenterprise collaboration (industry–industry) has remained absolutely dominant across the four phases from Phase I to Phase IV, with a notable trend of cross-regional division of labor within enterprise groups. Examples include BOE Technology Group Co., Ltd. (Hefei and Beijing branches), YITU Technology (Hangzhou and Guangzhou branches), and Shanghai Da’an Medical Laboratory (with its subsidiaries in Guangzhou, Chengdu, Zhuhai, and other cities). These cases reveal the strategic layout of large tech enterprises to integrate national resources through their internal innovation networks. This headquarters-branch collaboration model has enabled core cities in the YRD to transcend geographical distances through enterprise organizational boundaries and rapidly mobilize internal innovative elements of the group in response to sudden public health emergencies such as the COVID-19 pandemic.

**Table 3:** External innovation collaboration network of patent applicants in the YRD.

Phase	Applicant 1	Applicant 2	Weight
I	Hefei BOE Optoelectronics Technology Co., Ltd.	BOE Technology Group Co., Ltd.	8
	Jiadong Health Technology (Wuhu) Co., Ltd.	Jiadong Health Sports Technology (Shenzhen) Co., Ltd.	6
	Hangzhou YITU Network Technology Co., Ltd.	Guangzhou YITU Medical Technology Co., Ltd.	5
	Hangzhou YITU Medical Technology Co., Ltd.	Guangzhou YITU Medical Technology Co., Ltd.	5
	BOE Optoelectronics Technology Co., Ltd.	BOE Technology Group Co., Ltd.	5
	Duke Kunshan University	The Third Affiliated Hospital of Sun Yat-sen University (Sun Yat-sen University Liver Disease Hospital)	4
	Nanjing Renkang Hospital Co., Ltd.	Shenyang Shenbei Shenyi Hospital	4
	Suzhou Humeds Health Technology Co., Ltd.	Henan Butianshi Technology Co., Ltd.	4
	Suzhou Miteciser Artificial Intelligence Co., Ltd.	Henan Butianshi Technology Co., Ltd.	4
	China Mobile (Hangzhou) Information Technology Co., Ltd.	China Mobile Communications Group Co., Ltd.	4
II	Zhejiang Tsinghua Flexible Electronics Technology Research Institute	Tsinghua University	23
	Jiangsu Zhengxin Intelligent Technology Co., Ltd.	Shandong Zhengxin Medical Technology Co., Ltd.	12
	Migu Interactive Entertainment Co., Ltd.	Migu Culture Technology Co., Ltd.	9
	Hangzhou Jizhi Medical Technology Co., Ltd.	Beijing Litek Medical Technology Co., Ltd.	6
	Nanjing Shenqiao Medical Device Co., Ltd.	Zhengzhou Shenqiao Medical Device Co., Ltd.	6
	Hangzhou Jizhi Medical Technology Co., Ltd.	Beijing Litek Technology Co., Ltd.	5
	Hefei University of Technology	China Astronaut Research and Training Center	5
	Hefei Xinsheng Optoelectronics Technology Co., Ltd.	BOE Technology Group Co., Ltd.	5
	Shanghai DAAN Medical Laboratory Co., Ltd.	Chengdu Gaoxin DAAN Medical Laboratory Co., Ltd.	5
	Shanghai DAAN Medical Laboratory Co., Ltd.	Guangzhou DAAN Medical Testing Center Co., Ltd.	5
	Shanghai DAAN Medical Laboratory Co., Ltd.	Yunkang Health Industry Group Co., Ltd.	5
	Shanghai DAAN Medical Laboratory Co., Ltd.	Yunkang Health Industry Investment Co., Ltd.	5
	Zhenhe Precision Medical Laboratory (Wuxi) Co., Ltd.	Zhenhe (Beijing) Biotechnology Co., Ltd.	5
III	Shanghai Neusoft Medical Technology Co., Ltd.	Neusoft Medical Systems Co., Ltd.	31
	Fudan University	Zhuhai Fudan Innovation Research Institute	17
	Shanghai Airdoc Medical Technology Co., Ltd.	Beijing Airdoc Technology Development Co., Ltd.	15
	Duke Kunshan University	The Third Affiliated Hospital of Sun Yat-sen University	10
	Dianqi Biomedical Technology (Suzhou) Co., Ltd.	Dianqi Biomedical Technology (Beijing) Co., Ltd.	9
	Hefei BOE Optoelectronics Technology Co., Ltd.	BOE Technology Group Co., Ltd.	9
	Shanghai Jiao Tong University	Tsinghua University	9
	Aitang (Suzhou) Electronic Technology Co., Ltd.	Beijing Zhongqi Huakang Technology Development Co., Ltd.	8
	Ping An Zhennian (Shanghai) Enterprise Management Co., Ltd.	Ping An Technology (Shenzhen) Co., Ltd.	8
	Shanghai Boling Robot Technology Co., Ltd.	Tsinghua University	7
	Inventec (Shanghai) Technology Co., Ltd.	Inventec (Nanchang) Technology Co., Ltd.	7
	Zhejiang Ruihua Kangyuan Technology Co., Ltd.	Chengdu Ruihua Kangyuan Technology Co., Ltd.	7
IV	Hangzhou Deepwise Technology Co., Ltd.	Beijing Deepwise AI Technology Co., Ltd.	57
	CCB FinTech Co., Ltd.	China Construction Bank Corporation	13
	Midea Group (Shanghai) Co., Ltd.	Midea Group Co., Ltd.	13
	Wutong Sensing (Shanghai) Robotics Co., Ltd.	Wutong Sensing (Beijing) Technology Co., Ltd.	13
	iFlytek Co., Ltd.	Tsinghua University	12
	Hangzhou Calorie Sports Co., Ltd.	Beijing Calorie Technology Co., Ltd.	11
	Jiangsu Mandala Software Co., Ltd.	Jiangxi Mandala Software Co., Ltd.	11
	Kemei Boyang Diagnostic Technology (Shanghai) Co., Ltd.	Kemei Diagnostic Technology Co., Ltd.	10
	Hefei Hualing Co., Ltd.	Midea Group Co., Ltd.	9
	Hefei Midea Refrigerator Co., Ltd.	Midea Group Co., Ltd.	9
	Nanjing Jinfa Health Industry Co., Ltd.	Beijing Jinfa Technology Co., Ltd.	9
	VeriSilicon Microelectronics (Nanjing) Co., Ltd.	VeriSilicon Microelectronics (Hainan) Co., Ltd.	9
	VeriSilicon Microelectronics (Shanghai) Co., Ltd.	VeriSilicon Microelectronics (Hainan) Co., Ltd.	9
	China Mobile (Suzhou) Software Technology Co., Ltd.	China Mobile Communications Group Co., Ltd.	9

When the microlevel network of patent applicants is compared with the external collaborative network of YRD cities shown in Figure 3, it is evident that Beijing leads significantly in the rankings of external collaborative innovation cities for the YRD. The microfoundations of this leadership are the high-frequency collaborative innovations between numerous Beijing-based innovation entities, such as BOE Technology, Deepwise AI, Airdoc Technology, China Mobile, Tsinghua University, and the Cancer Hospital of the Chinese Academy of Medical Sciences, and their YRD branches. For instance, the collaboration intensity between Hangzhou Deepwise AI and Beijing Deepwise AI reached 57 in Phase IV, and that between Shanghai Airdoc Medical and Beijing Airdoc Technology stood at 15 in the same phase. These partnerships collectively support Beijing’s absolute status as the primary external collaborative city for the YRD. The increase in rankings for cities such as Guangzhou, Shenzhen, Shenyang, Zhuhai, and Foshan can also be explained at the micro level. Guangzhou’s high ranking is driven by collaboration between Guangzhou Yitu and Hangzhou Yitu, Sun Yat-sen Memorial Hospital and Duke Kunshan University, and Da’an Medical Laboratory with its Shanghai counterpart. The collaborative link with a weight of 31 between Neusoft Medical Shenyang and Shanghai branches in Phase III directly corresponds to Shenyang’s abnormal jump in ranking to the top five in that phase. The multiple collaborative and innovative ties between Foshan Midea Group and its counterparts in Shanghai and Hefei provide a detailed explanation for Foshan’s rise into the top four in Phase IV. Additionally, cities such as Nanchang and Haikou have begun to enter the YRD’s external collaboration field through cross-regional linkages with companies such as Wuxi Mandala Technology and Nanjing VeriSilicon Microelectronics, reflecting a continuing trend of external collaborative innovation networks diffusing into broader regions.

The internal networks of large enterprise groups are not only the outcome of corporate strategic layout but also a crucial explanatory force for the evolution of regional innovation spatial patterns. In traditional innovation geography, geographic proximity among cities is the primary prerequisite for technological collaboration. However, this study reveals that major enterprise groups, including BOE, Deepwise AI, Midea, and Yuwell Medical, have overcome the geographical distance barrier and established high-intensity collaborative innovation ties between core cities in the YRD and external cities such as Beijing, Guangzhou, Shenzhen, and Foshan by adopting a headquarters-branch model of the internal cross-regional division of labor.

### 3.6 Spatial pattern of the internal collaborative innovation network in the YRD

**Figure 4:**
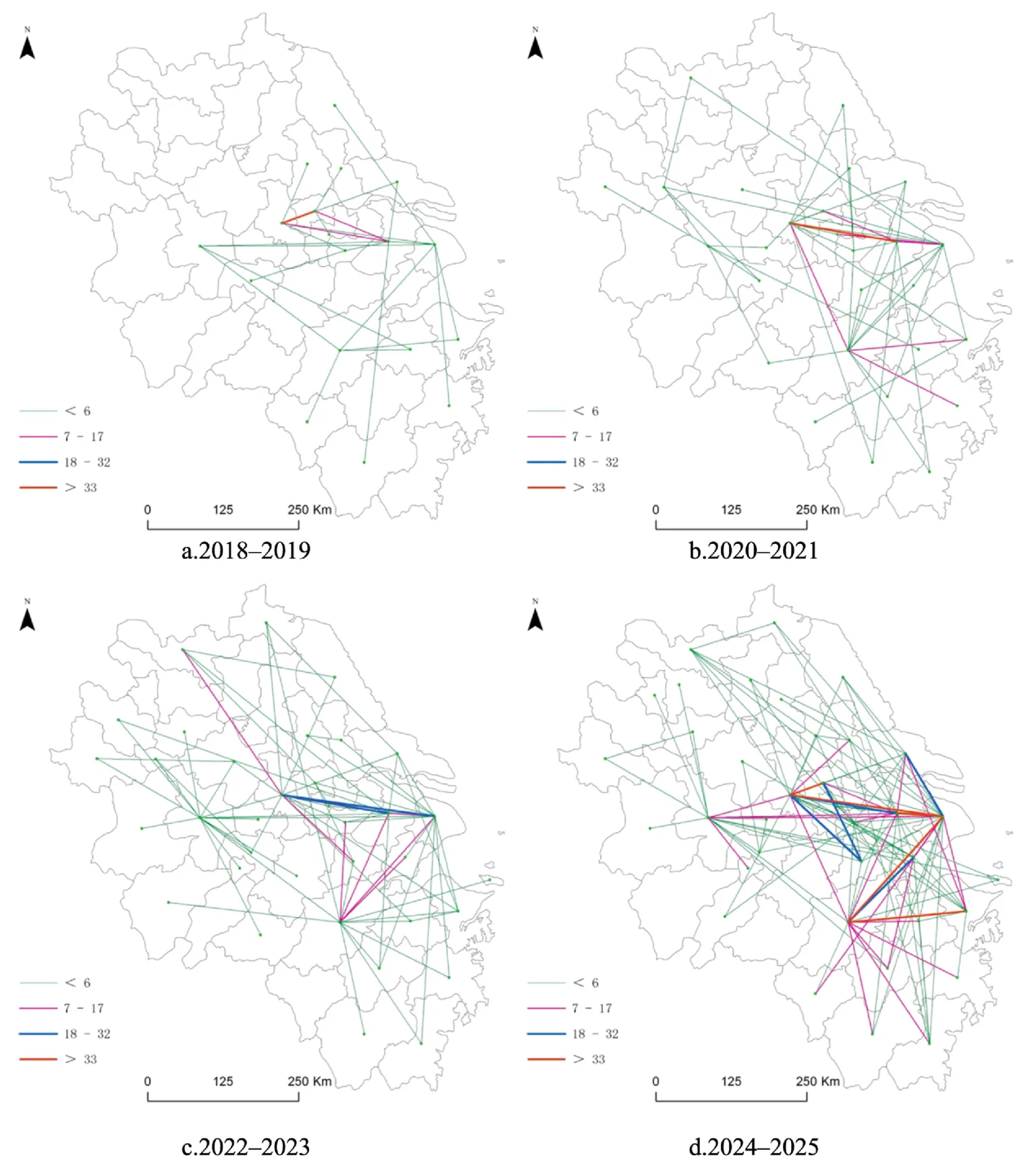
Internal collaborative innovation network in the YRD.

From the perspective of internal collaborative innovation within the YRD (Figure 4), the first-tier collaborative innovation in the fourth phase is entirely concentrated among the five core cities of Shanghai, Suzhou, Nanjing, Hangzhou, and Ningbo. High-weighted innovation paths, such as Shanghai–Suzhou (59), Nanjing–Zhenjiang (45), and Hangzhou–Ningbo (43), constitute the backbone of internal innovation in the YRD. This second-tier collaborative innovation reflects the strong radiation from core cities to peripheral cities. Examples include Huzhou–Zhenjiang (32), Huzhou–Nanjing (29), Hangzhou–Jiaxing (28), Nantong–Shanghai (24), and Nanjing–Suzhou (21). This marks a shift in which AI-driven medical manufacturing and interdisciplinary engineering technologies are spreading from research and development hubs to outlying areas, driving vertical specialization within the regional public health technology innovation chain. Fourth-tier collaborative innovation covers all 41 cities in the YRD, although peripheral cities in northern Anhui and northern Jiangsu, including Chizhou, Bozhou, Suzhou, and Huaibei, have relatively low link weights, which have all been incorporated into the YRD’s innovation network.

In terms of the dynamic evolution of the spatial pattern, innovation links in Phase I were highly concentrated between Nanjing and Zhenjiang, with low participation from surrounding cities. Entering Phase II, innovation ties among cities along the Shanghai–Suzhou–Nanjing axis were significantly strengthened. In Phase III, Shanghai’s status as a central hub was further highlighted, establishing high-intensity innovation links with Suzhou, Nanjing, and Hangzhou. Moreover, Hefei began to exert radiation and influencing effects on central Anhui cities such as Huainan, Chuzhou, and Wuhu. This two-tiered, tiered-radiation mechanism has effectively promoted the joint construction and sharing of public health R&D infrastructure across provincial boundaries. In Phase IV, as a top-tier hub, Shanghai maintained high-weight collaborative innovation with Suzhou, Nanjing, Hangzhou, Ningbo, Nantong, and Jiaxing. Notably, Huzhou formed high-intensity innovation links with Nanjing and Zhenjiang in Phase IV that are far out of proportion to its economic scale, which may be attributed to the cross-regional layout of specific leading enterprises and the in-depth collaboration in major industry-university-research (IUR) projects. This phenomenon strongly demonstrates that, driven by policy guidance and public health needs, specific locations can achieve a significant leap forward and make rapid progress in public health R&D capabilities by strategically absorbing industrial spillovers from core cities.

From the micro perspective of the internal collaborative network of patent applicants in the YRD (Table 4), interenterprise collaboration (industry-industry) has remained absolutely dominant throughout the evolution across the four phases, with a prominent trend of cross-city division of labor within enterprise groups. The Yuwell Medical Cluster is taken as a typical example. In Phase I, Jiangsu Yuwell Medical Equipment Co., Ltd. (Zhenjiang) formed a radial network with Zhenjiang as the headquarters, with Nanjing and Suzhou as R&D and manufacturing bases, in collaboration with Nanjing Yuwell Software Technology Co., Ltd., Jiangsu Yuwell Information System Co., Ltd. (Nanjing), and Suzhou Medical Supplies Factory Co., Ltd. (Suzhou). Multiple high-weight collaborative innovation paths under this network collectively contributed to the major flow of internal collaboration in this phase. This pattern evolved into the bilateral strengthening of Nanjing–Suzhou and Zhenjiang–Suzhou links in Phase II and advanced to a triangular structure with tricity linkage in Phase IV, involving Jiangsu Yuwell Poctech Biotechnology Co., Ltd. (Zhenjiang), Jiangsu Yuekai Biotechnology Co., Ltd. (Nanjing), and Zhejiang Poctech Medical Device Co., Ltd. (Huzhou). The high-weight collaborative innovation ties of Zhenjiang–Nanjing (33), Zhenjiang–Huzhou (31), and Nanjing–Huzhou (21) together constitute a microcosmic model of internal innovative collaboration in the YRD in the post-COVID-19 era.

**Table 4:** Internal innovation connection network of patent applicants in the YRD.

Phase	Applicant 1	Applicant 2	Weight
I	Jiangsu Yuwell Medical Equipment Co., Ltd.	Nanjing Yuwell Software Technology Co., Ltd.	50
	Jiangsu Yuwell Medical Equipment Co., Ltd.	Jiangsu Yuwell Information System Co., Ltd.	17
	Jiangsu Yuwell Medical Equipment Co., Ltd.	Suzhou Medical Supplies Factory Co., Ltd.	12
	Suzhou Medical Supplies Factory Co., Ltd.	Jiangsu Yuwell Information System Co., Ltd.	5
	Suzhou Yuwell Medical Technology Co., Ltd.	Jiangsu Yuwell Information System Co., Ltd.	4
	Southeast University	Suzhou Madison Medical Technology Co., Ltd.	3
	Suzhou Yuwell Medical Technology Co., Ltd.	Jiangsu Yuwell Medical Equipment Co., Ltd.	3
	Nanjing Yuwell Software Technology Co., Ltd.	Jiangsu Yuwell Technology Development Co., Ltd.	2
	Ningbo Hyk Smart Technology Co., Ltd.	Zhejiang University	2
	Suzhou Medical Supplies Factory Co., Ltd.	Jiangsu Yuwell Technology Development Co., Ltd.	2
II	Jiangsu Yuwell Medical Equipment Co., Ltd.	Suzhou Yuwell Medical Technology Co., Ltd.	14
	Jiangsu Yuwell Information System Co., Ltd.	Suzhou Yuwell Medical Technology Co., Ltd.	11
	Nanjing Yuwell Software Technology Co., Ltd.	Suzhou Yuwell Medical Technology Co., Ltd.	8
	Zhongda Hospital Southeast University	Suzhou Meddatas Medical Technology Co., Ltd.	6
	Jiangsu Yuwell Information System Co., Ltd.	Suzhou Medical Supplies Factory Co., Ltd.	6
	Nanjing Yuwell Software Technology Co., Ltd.	Suzhou Medical Supplies Factory Co., Ltd.	5
	Zhejiang Geely Automobile Research Institute Co., Ltd.	Zhejiang Geely Holding Group Co., Ltd.	4
	MCC Huatian Engineering Technology Co., Ltd.	MCC Huatian Nanjing Engineering Technology Co., Ltd.	4
	Anhui University of Science and Technology	Hefei Boxie Electronic Technology Co., Ltd.	3
	Chuzhou Polytechnic	Jiangsu University	3
	Geely Automobile Research Institute (Ningbo) Co., Ltd.	Zhejiang Geely Holding Group Co., Ltd.	3
	Nanjing Guoke Medical Engineering Technology Development Co., Ltd.	Suzhou Institute of Biomedical Engineering and Technology, Chinese Academy of Sciences	3
	The Ninth People’s Hospital Affiliated to Shanghai Jiao Tong University School of Medicine	Suzhou Institute of Biomedical Engineering and Technology, Chinese Academy of Sciences	3
	Zhejiang University	Xianju Aisheng Medical Technology Co., Ltd.	3
	Focusight (Jiangsu) Technology Co., Ltd.	Focusight Intelligent Technology (Jiangsu) Co., Ltd.	3
III	Zhejiang Poctech Medical Device Co., Ltd.	Jiangsu Yuekai Biotechnology Co., Ltd.	11
	Suzhou Yuwell Medical Technology Co., Ltd.	Nanjing Yuwell Software Technology Co., Ltd.	9
	Suzhou Medical Supplies Factory Co., Ltd.	Nanjing Yuwell Software Technology Co., Ltd.	7
	Wuxi Qing’er Huasheng Technology Co., Ltd.	Hangzhou Erqingcong Technology Co., Ltd.	7
	Hangzhou Hewei Technology Development Co., Ltd.	Aitang (Suzhou) Electronic Technology Co., Ltd.	6
	Fudan University	Yiwu Research Institute, Fudan University	5
	Nanjing Pinsheng Medical Technology Co., Ltd.	Shanghai Untangled Biotechnology Co., Ltd.	5
	Nanjing Yunlian Shuke Technology Co., Ltd.	Shanghai Juyin Information Technology Co., Ltd.	5
	Hefei University of Technology	Chuzhou Yiran Sensing Technology Research Institute Co., Ltd.	4
	Ningbo Institute of Artificial Intelligence, Shanghai Jiao Tong University	Ningbo Industrial Internet Research Institute Co., Ltd.	4
	Zhejiang University	Jiaxing Research Institute, Zhejiang University	4
IV	Jiangsu Yuwell Poctech Biotechnology Co., Ltd.	Jiangsu Yuekai Biotechnology Co., Ltd.	33
	Jiangsu Yuwell Poctech Biotechnology Co., Ltd.	Zhejiang Poctech Medical Device Co., Ltd.	31
	Jiangsu Yuekai Biotechnology Co., Ltd.	Zhejiang Poctech Medical Device Co., Ltd.	21
	Zhejiang Zeekr Intelligent Technology Co., Ltd.	Zhejiang Geely Holding Group Co., Ltd.	17
	Zhiben Medical Technology (Shanghai) Co., Ltd.	Shanghai Zhiben Clinical Laboratory Co., Ltd.	12
	Shanghai Shende Non-Invasive Era Medical Technology Co., Ltd.	Nantong Shende Medical Device Technology Co., Ltd.	11
	Jiangsu Sysdiagno Biotechnology Co., Ltd.	Sysdiagno (Nanjing) Biotechnology Co., Ltd.	10
	Nantong Shende Medical Device Technology Co., Ltd.	Shende (Ningbo) Medical Device Technology Co., Ltd.	10
	Shanghai Shende Medical Technology Co., Ltd.	Shende (Ningbo) Medical Device Technology Co., Ltd.	10
	VeriSilicon Microelectronics (Nanjing) Co., Ltd.	VeriSilicon Microelectronics (Shanghai) Co., Ltd.	10
	Zhejiang Tsinghua Flexible Electronics Technology Research Institute	Qiantang Science and Technology Innovation Center	10

A comparison between the microlevel network at the patent applicant level and the internal collaboration network of the YRD in Figure 4 enables a deeper understanding of the formation mechanism of the macro spatial pattern. The ultrahigh-weight collaborative innovation link of Nanjing–Zhenjiang (72) in Phase I is microfounded on the intensive internal collaboration among multiple entities of Yuwell Medical in Nanjing and Zhenjiang. The emergence of Nanjing–Suzhou (43) as the strongest collaborative innovation link in Phase II corresponds to the interbranch collaboration of Yuwell Medical in Nanjing and Suzhou, as well as the IUR collaboration between Zhongda Hospital Southeast University and Suzhou-based enterprises. The sudden rise of the Huzhou-Nanjing (11) link in Phase III is clearly matched at the micro level by the collaboration between Zhejiang Poctech Medical Device Co., Ltd. (Huzhou) and Jiangsu Yuekai Biotechnology Co., Ltd. (Nanjing). The sharp rise to high ranks of Huzhou–Zhenjiang (32) and Huzhou–Nanjing (29) in Phase IV is directly underpinned by the three high-weight collaborative innovation paths (33, 31, 21) of Yuwell Medical’s Zhenjiang–Huzhou–Nanjing triangle (see Table 5).

**Table 5:** Factors influencing geodetector analysis results in the YRD.

Dimension	Influencing factors	Intensity of internal collaborative innovation						Intensity of external collaborative innovation					
		2018-2019		2020—2021		2022—2023		2018-2019		2020—2021		2022—2023	
		Q value	*p* value	Q value	*p* value	Q value	*p* value	Q value	*p* value	Q value	*p* value	Q value	*p* value
Economic development level	Secondary and tertiary industry output value to GDP	0.3096	0.0281	0.4822	0.0000	0.4045	0.0043	0.3095	0.0281	0.3913	0.0058	0.3365	0.0161
	per capita GDP	0.4313	0.0043	0.5292	0.0000	0.3246	0.0362	0.4194	0.0055	0.4728	0.0036	0.4294	0.0059
	regional GDP	0.6050	0.0000	0.6497	0.0026	0.7414	0.0000	0.8520	0.0000	0.7073	0.0000	0.8931	0.0000
	Total retail sales of consumer goods	0.5602	0.0058	0.8002	0.0000	0.8587	0.0000	0.8455	0.0000	0.8946	0.0000	0.9237	0.0000
	GDP growth rate	0.0275	0.7971	0.1499	0.1262	0.2553	0.0947	0.0249	0.8237	0.0863	0.3555	0.3342	0.0307
	Total profits of industrial enterprises above a designated size	0.3274	0.3888	0.5966	0.0032	0.6034	0.0026	0.5545	0.0928	0.5605	0.0052	0.7917	0.0000
Innovation factor support	Science expenditure	0.4843	0.0383	0.6443	0.0025	0.7687	0.0000	0.8855	0.0000	0.7263	0.0000	0.8427	0.0000
	Education expenditure	0.5102	0.0088	0.6411	0.0033	0.7101	0.0000	0.6625	0.0000	0.6794	0.0025	0.8656	0.0000
	Number of full-time teachers in regular higher education institutions	0.4992	0.0216	0.7783	0.0000	0.8562	0.0000	0.7464	0.0000	0.9073	0.0000	0.8893	0.0000
	Number of enrolled students in regular higher education institutions	0.6509	0.0111	0.6079	0.0139	0.7681	0.0000	0.9495	0.0000	0.7956	0.0000	0.7679	0.0000
	Number of regular higher education institutions	0.5488	0.0256	0.7329	0.0000	0.8141	0.0000	0.9491	0.0000	0.8115	0.0000	0.8246	0.0000
	Postal and telecommunications service revenue	0.4399	0.0557	0.5500	0.0084	0.6111	0.0093	0.8161	0.0000	0.6507	0.0000	0.7320	0.0000
Fiscal and financial support	Local government general budget expenditure	0.5099	0.0088	0.6890	0.0000	0.6149	0.0077	0.6655	0.0000	0.7358	0.0000	0.7286	0.0000
	Year-end balance of various loans of financial institutions	0.6221	0.0000	0.7193	0.0000	0.8574	0.0000	0.8627	0.0000	0.7605	0.0000	0.9325	0.0000
	Year-end balance of various deposits of financial institutions	0.5804	0.0035	0.8163	0.0000	0.8174	0.0000	0.8455	0.0000	0.9021	0.0000	0.9320	0.0000
Openness level	Number of foreign-invested enterprises	0.3676	0.2734	0.6342	0.0139	0.6211	0.0197	0.6326	0.0188	0.6555	0.0055	0.8175	0.0000

Shanghai’s central position in the internal network is also supported by microlevel evidence. In Phase III, multiple IUR collaborative innovation ties exist between Shanghai and Nanjing, Suzhou, and Ningbo. In Phase IV, the collaborative links with weights of 10 and 11 between Shende Medical Technology (Shanghai) Co., Ltd., and its branches in Nantong and Ningbo, the 8-weight link between VeriSilicon Microelectronics (Nanjing) Co., Ltd., and its Shanghai headquarters, and the 8-weight link between Bewatec (Zhejiang) Medical Equipment Co., Ltd., and its Shanghai subsidiary. These collaborations collectively form the microcosmic cornerstone of Shanghai as an innovation hub in the YRD.

## 4 Influencing factor analysis

### 4.1 Factor selection

On the basis of existing scholarly research, the influencing factors of AI medical device innovation networks are closely related to a city’s innovation level, economic development level, and local fiscal capacity. Given this, and because the statistical yearbook data for the relevant cities in Phase IV have not yet been updated, this study takes the external and internal connection intensities of cities in the YRD from Phases I to III as the dependent variables. A higher weighted degree centrality indicates that a city possesses a greater volume of direct innovation resources within the network and maintains more frequent technological collaborations with external actors. Such direct collaborative ties enhance a city’s capacity to access and mobilize innovation resources, thereby strengthening its ability to rapidly organize technological innovation, facilitate industrial collaboration, and accelerate the commercialization of medical devices in response to routine healthcare needs and public health emergencies. Therefore, compared with betweenness centrality, which emphasizes brokerage roles, and closeness centrality, which reflects network accessibility, weighted degree centrality is more consistent with the objective of this study, namely, measuring the intensity of collaborative innovation and the spatial allocation of innovation resources.

According to the Regional Innovation System theory, regional innovation capability arises from the interaction among industrial foundations, knowledge creation, institutional support, and external linkages (70). Given that the AI medical device industry is characterized by knowledge intensity, capital intensity, and multi-actor collaborative innovation, the formation and evolution of its innovation networks depend on the agglomeration, allocation, and diffusion of regional innovation resources. Accordingly, explanatory variables were selected from four complementary dimensions: economic development, innovation resource support, fiscal and financial support, and openness. Specifically, the economic development dimension was represented by the proportion of secondary and tertiary industry output value to GDP, per capita GDP, regional GDP, total retail sales of consumer goods, GDP growth rate, and total profits of industrial enterprises above a designated size are selected as independent variables. In terms of innovation factor support, science expenditure, education expenditure, the number of full-time teachers in regular higher education institutions, the number of students enrolled in regular higher education institutions, the number of regular higher education institutions, and postal and telecommunications service revenue are selected as independent variables. In terms of fiscal and financial support, local government general budget expenditure, the year-end balances of various financial institutions’ loans, and the year-end balances of various financial institutions’ deposits are selected as independent variables. Regarding the degree of openness, the number of foreign-invested enterprises is selected as the independent variable.

### 4.2 Analysis results

Overall, the Geodetector results indicate that the explanatory power of most variables increased over time, suggesting that the spatial differentiation of innovation collaboration became increasingly associated with regional innovation resources and economic conditions following the COVID-19 pandemic (see Table 5). Nevertheless, considerable heterogeneity exists among the explanatory variables, reflecting the diverse mechanisms underlying the evolution of the innovation network.

From the perspective of stable explanatory factors, indicators representing economic scale, innovation resources, and financial support consistently exhibited strong explanatory power throughout the three phases. Within the economic development dimension, regional GDP and total retail sales of consumer goods remained among the strongest explanatory variables for both internal and external collaboration intensity, with q-values generally exceeding 0.70 in the later stages. This suggests that the formation of innovation collaboration networks depends primarily on cities’ long-term economic strength rather than short-term economic fluctuations. Cities with larger economic scales not only possess stronger industrial foundations and greater market demand but also provide abundant application scenarios and commercialization opportunities for AI medical device technologies, thereby continuously attracting collaborative innovation activities.

A similar pattern is observed for the innovation resource support dimension. Indicators related to higher education, including the number of higher education institutions, the number of full-time university teachers, and university enrollment, as well as science expenditure, consistently exhibit relatively high explanatory power. In particular, the explanatory power of the number of full-time university teachers and higher education institutions continued to increase, with q-values exceeding 0.80 during the post-pandemic period. This finding reflects the knowledge-intensive nature of the AI medical device industry. The continuous accumulation of scientific talent, interdisciplinary knowledge, and public R&D investment provides the essential conditions for sustained collaboration among universities, hospitals, research institutes, and enterprises. Compared with physical infrastructure, knowledge resources constitute a more fundamental determinant of the spatial organization of innovation networks.

Financial support also represents one of the most stable associated factors. The balances of financial institutions’ loans and deposits consistently ranked among the variables with the highest explanatory power, particularly for external collaboration intensity, with q-values exceeding 0.93 in Phase III. This indicates that adequate financial resources enhance cities’ capacity to sustain long-term R&D investment, absorb innovation risks, and establish cross-regional collaborative relationships. The relatively stable explanatory power across different stages further suggests that financial support serves as a long-term institutional foundation for innovation collaboration rather than a short-term policy stimulus.

In contrast, several variables exhibit pronounced dynamic characteristics. The explanatory power of GDP growth, total profits of industrial enterprises above the designated size, and the number of foreign-invested enterprises increased substantially following the COVID-19 pandemic. Among these, GDP growth became statistically significant during Phase III, indicating that cities’ economic recovery capacity gradually emerged as an important factor of collaborative innovation. A plausible explanation is that before and during the COVID-19 pandemic, intercity collaboration was primarily sustained by existing innovation linkages and crisis-driven cooperation rather than differences in regional economic growth. As economic activities recovered following the major COVID-19 shock, regional growth increasingly shaped cities’ capacity to attract innovation resources and establish new collaborative relationships. Similarly, the explanatory power of the number of foreign-invested enterprises increased from an insignificant level before the pandemic to above 0.80 for external collaboration in Phase III, suggesting that openness exerts a lagged effect on innovation collaboration. As international technological exchanges gradually resumed after the pandemic, cities with stronger international linkages recovered more rapidly and became better positioned to attract external innovation resources. These findings indicate that while structural innovation capacity determines cities’ long-term competitiveness, economic recovery momentum and openness show increasingly strong associations with collaborative innovation.

A noteworthy finding is that almost all explanatory variables exhibit stronger explanatory power for external collaboration intensity than for internal collaboration intensity. For example, regional GDP, financial institution deposits and loans, university resources, and the number of foreign-invested enterprises all achieve substantially higher q-values in the external collaborative network. This suggests that cross-regional collaboration places higher demands on cities’ comprehensive innovation capacity than collaboration within the YRD, where geographical proximity, institutional integration, and established innovation linkages reduce collaboration barriers. From the perspective of absorptive capacity theory, cities with stronger university resources, greater science expenditure, and richer innovation infrastructure are better able to identify, assimilate, and exploit external knowledge, thereby facilitating cross-regional collaboration. By contrast, economic development and financial resources primarily provide the industrial and institutional foundations for local innovation agglomeration. Therefore, while economic strength supports regional clustering, innovation resources exhibit stronger explanatory power for embedding cities into broader interregional knowledge networks, highlighting the importance of knowledge infrastructure in promoting cross-regional technology diffusion.

These findings are also consistent with the persistent core–periphery structure identified in the network analysis. The consistently high explanatory power of economic scale, university resources, and financial support suggests that cities possessing advantages in these dimensions are more likely to occupy central positions within the collaboration network and continuously attract innovation resources from surrounding cities. Meanwhile, the increasing explanatory power of openness and economic recovery following the major COVID-19 shock indicates the evolution of the innovation network appears to be increasingly associated with a broader innovation ecosystem integrating knowledge production, financial support, and external connectivity.

## 5 Conclusions and implications

### 5.1 Conclusion

This study takes the YRD to explore the mechanism by which firm collaboration networks drive technology diffusion and shape regional development disparities in the AI medical device industry. Integrating SNA with the Geodetector method, this research analyzes the evolutionary characteristics of innovation networks from a multi-dimensional perspective against the dual backdrop of digital development and external shocks.

First, the continuous expansion of the innovation network scale and the accelerated development of the bipartite network indicate that firms in the YRD are deeply integrating into the national AI medical innovation system. In the post-pandemic period, both the weighted degree and clustering coefficient of the network have shown an upward trend, reflecting the growing tightness and cohesion of inter-firm collaboration, the enhanced resilience of the regional healthcare innovation system, and the improved responsiveness of technology diffusion in crisis scenarios.

Second, the innovation network exhibits a distinct hub-and-spoke structural characteristic both inter-regionally and intra-regionally. Core cities represented by Shanghai, together with leading industrial firms, have jointly become strategic hubs for promoting cross-regional knowledge flows, while emerging cities are gradually integrating into this network system. At the micro level, inter-firm collaborative behaviors, especially the internal coordination of cross-regional enterprise groups and the in-depth integration of industry, academia, and research institutions, play a decisive role in the evolution of the innovation network. These findings indicate that eliminating collaborative barriers among micro-level entities is essential to improving the operational efficiency and inclusiveness of medical innovation networks.

Third, the results show that the level of economic development and fiscal and financial support exert a significant impact on the formation and evolution of collaboration networks, while innovation-related resources play an especially important role in the construction of firms’ external linkages. The influence of economic growth has become even more prominent following the major COVID-19 shock, which means that the momentum of economic recovery can effectively stimulate the vitality of collaborative innovation. However, the uneven distribution of collaboration intensity across cities also reflects the persistent existence of structural constraints such as institutional fragmentation, which not only limit the network participation of peripheral regions but also serve as an important contributor to the formation of regional development disparities.

### 5.2 Theoretical contributions

(1) In this study, an analytical framework for the technological innovation network of the AI medical device industry in the YRD is constructed from the dual perspectives of national and local scales, overcoming the limitations of single-scale research. It reveals the differences in evolutionary paths between the bipartite network and the local network and provides a new analytical paradigm for understanding the complex spatial structure of regional innovation networks.

(2) By incorporating the COVID-19 pandemic as a temporal context in the analysis, this study reveals the evolutionary characteristics of the AI medical device technological innovation network at different phases, especially the significant improvement in the internal network following the major COVID-19 shock. It provides empirical evidence for understanding the possible response mechanisms of innovation systems under external shocks.

(3) This study combines the analysis of macro network patterns with the analysis of micro patent applicant collaboration, opening up the analytical channel from the urban level to the organizational level. From typical cases such as Yuwell Medical, BOE, and Deepwise AI, it extracts micro mechanisms such as cross-regional integration within enterprise groups and in-depth integration of IUR, and provides a more solid explanation from the perspective of organizational behavior for the formation of the spatial structure of innovation networks.

### 5.3 Implications for public health

(1) The findings indicate that Shanghai, Suzhou, Nanjing, and Hangzhou occupy pivotal positions in both the internal and external collaborative networks, whereas most cities in Anhui Province and several cities in northern Jiangsu exhibit relatively weak external collaboration intensity. To enhance regional resilience in responding to major public health emergencies, the YRD should strengthen the coordinating role of these core cities while promoting the diffusion of high-quality innovation resources to peripheral cities through targeted policy support. Such measures would contribute to a more balanced distribution of AI medical device innovation capacity across the region.

(2) The observed cross-regional division of labor within AI medical device enterprise groups, together with the deep integration of industry–university–hospital collaboration, suggests that governments should encourage leading enterprises to establish cross-regional innovation networks and further support universities and hospitals in technology development. This collaborative model enables innovation activities to transcend geographical constraints through organizational networks, allowing innovation resources to be rapidly mobilized across regions during major public health emergencies.

(3) The Geodetector results demonstrate that strong financial support and well-developed information infrastructure are key factors enhancing the resilience of AI medical device innovation networks. Therefore, policymakers should continue to strengthen the financial hub functions of core cities while increasing investment in digital and information infrastructure in peripheral cities. Such efforts would improve the continuity and stability of cross-regional innovation collaboration during future public health emergencies.

### 5.4 Research limitations and prospects

(1) Due to limitations in data availability, this study uses the patent collaboration network as a proxy for regional public health innovation capacity and has not yet incorporated other measures of innovation linkages, such as technology transfer, talent mobility, and capital investment, or other indicators of public health governance capacity. Therefore, the characterization of innovation networks and public health innovation capacity remains somewhat limited. Future research could integrate multi-source data, including patents, academic papers, talent, and capital, to construct a more comprehensive framework for analyzing innovation networks.

(2) Due to limitations in data completeness, Geodetector was applied only to Phases I through III. Furthermore, this study employed only a phase-based analytical framework and excluded intra-city collaborative patents. These design choices may underestimate the contribution of local innovation ecosystems and limit the ability to capture annual network dynamics and assess the long-term impacts of the COVID-19 pandemic. Future research could incorporate more up-to-date post-pandemic data, annual network analysis, and multi-level collaboration networks to gain a more comprehensive understanding of the long-term evolution of AI medical device innovation networks and their explanatory mechanisms.

(3) The Geodetector cannot further distinguish whether the changes stem from economic recovery, policy interventions, or adjustments to inter-city collaboration strategies. Future research could combine panel econometric models or causal inference methods with dynamic network analysis to further clarify these mechanisms.

(4) This study focuses only on the YRD and does not include a comparative analysis of other coastal urban agglomerations, making it difficult to judge the uniqueness and universality of the evolutionary model of the YRD innovation network. In the future, comparative research on urban agglomerations such as the PRD and the BTH can be conducted to explore similarities and differences in the evolution of innovation networks across regions and the causes behind them.

## Data Availability

The raw data supporting the conclusions of this article will be made available by the authors, without undue reservation.

## Footnotes

1
https://www.nmpa.gov.cn/xxgk/fgwj/gzwj/gzwjylqx/20260224172356196.html

2
https://www.shkjdw.gov.cn/c/2020-02-05/519956.shtml

3
https://www.gov.cn/zhengce/2019-12/01/content_5457442.htm
